# MRI-based assessment of greater tuberosity exposure in massive rotator cuff tears: a systematic review of current evidence and methodological gaps

**DOI:** 10.1186/s12891-026-10705-3

**Published:** 2026-11-03

**Authors:** Jan Herrmann, Jonas Limmer, David Neubauer, Thilo Lehmeyer, Jan Oberfeld, Maximilian Rudert, Kilian List, Felix Hochberger

**Affiliations:** Department of Orthopaedic Surgery, Julius-Maximilians University, Koenig-Ludwig-Haus, Brettreichstrasse 11, 97074 Wuerzburg, Wuerzburg, Germany

**Keywords:** Massive rotator cuff tear, MRI, Greater tuberosity exposure, Irreparability, Fatty infiltration, Tendon retraction, Structural imaging

## Abstract

**Background:**

Massive rotator cuff tears (MRCTs) lack a standardized and reproducible imaging-based definition. A recent Delphi consensus introduced greater tuberosity exposure (GTE) as a defining criterion for MRCTs, proposing that MRCTs be classified by either involvement of at least two rotator cuff tendons or ≥ 67% GTE on magnetic resonance imaging (MRI).

**Purpose:**

To evaluate the role of GTE in the MRI-based definition and structural characterization of MRCTs and to describe the extent and methodological maturity of the evidence available for GTE and established MRI-derived parameters.

**Methods:**

A systematic literature search was conducted in PubMed/MEDLINE, Embase, and the Cochrane Central Register of Controlled Trials from database inception to February 17, 2026, and updated on October 9 and 11, 2026 with supplementary citation searching, in accordance with PRISMA 2020 guidelines. Studies were included if they investigated MRCTs using MRI-based or MRI-transferable imaging and reported structural definitions, measurement methods, or associations with irreparability, structural healing, or clinical outcomes. Prognostic studies were assessed using the Quality In Prognosis Studies (QUIPS) tool. Findings were synthesized narratively; no meta-analysis was performed due to substantial heterogeneity in study designs, imaging protocols, and outcome definitions. The synthesis described the extent and methodological maturity of the available evidence and did not compare diagnostic or prognostic performance between parameters.

**Results:**

A total of 34 studies were included. No study evaluated GTE as a quantitative MRI-derived parameter. Only two studies addressed footprint coverage on imaging, one qualitatively and one by a semi-quantitative five-zone score of residual tendon attachment; neither measured GTE as a defined exposure parameter or applied predefined thresholds. In contrast, MRCTs were consistently characterized using indirect structural parameters, primarily tendon involvement and tear size. Established MRI-derived variables—including tear size, tendon retraction, fatty infiltration, muscle atrophy, and superior humeral migration—were assessed using established measurement frameworks based on defined anatomical landmarks, notwithstanding methodological heterogeneity in imaging protocols and thresholds across studies. These parameters were frequently evaluated in relation to irreparability, structural healing, and functional outcomes, whereas no study evaluated a direct quantitative MRI-based measure of GTE in relation to a clinical or structural endpoint.

**Conclusion:**

No standardized and outcome-validated MRI-based method for quantifying GTE was identified within the scope of this review. Despite its conceptual appeal as a direct representation of footprint deficiency, GTE has not yet undergone standardized measurement development or outcome-based validation. The evidence base for established MRI-derived parameters is more extensive and methodologically mature, with operationalized measurement methods and reported outcome associations; this does not imply clinical inferiority of GTE. Future research should focus on developing reproducible MRI-based measurement techniques for GTE and evaluating its diagnostic and prognostic relevance in dedicated validation studies.

**Study design:**

Systematic review; Level of evidence, 3. Individual included studies ranged from Level II to Level V.

**Supplementary Information:**

The online version contains supplementary material available at https://doi.org/10.1186/s12891-026-10705-3.

## Introduction

Massive rotator cuff tears (MRCTs) represent a severe form of rotator cuff pathology characterized by advanced structural deterioration and substantial functional impairment [1]. Despite their clinical relevance, no universally accepted and consistently applied imaging-based definition of MRCTs has yet been established. Historically, MRCTs have been defined using heterogeneous structural criteria, including absolute tear size [2], involvement of two or more tendons [3], and geometric tear characteristics [4, 5]. These approaches differ in conceptual rationale, measurement methodology, and clinical applicability, contributing to considerable interstudy variability.

Magnetic resonance imaging (MRI) is the primary modality for structural assessment of MRCTs and forms the basis of most imaging-based descriptions of tear extent and morphology. Accordingly, several MRI-derived parameters are routinely used to characterize structural severity in both clinical practice and research. Recently, consensus-based criteria for MRCTs were proposed, including either involvement of at least two rotator cuff tendons or ≥ 67% greater tuberosity exposure (GTE) on MRI [6]. This definition represents a conceptual shift from absolute tear size toward relative footprint deficiency and introduces GTE as a potential structural marker of tear severity. Notably, the original Delphi consensus explicitly permitted the ≥ 67% GTE threshold to be assessed either by MRI or intraoperatively, with no single modality reaching independent consensus among the expert panel. The present review focuses specifically on the MRI-based quantification of this threshold, as intraoperative assessment does not constitute a reproducible, pre-treatment imaging biomarker. This review was designed to evaluate the body of evidence addressing GTE beyond this foundational Delphi consensus, which is discussed throughout this manuscript as the conceptual basis for the review question.

However, despite its incorporation into the consensus definition, GTE lacks methodological standardization and clinical validation. In contrast, several established MRI-derived variables have been extensively investigated in relation to structural severity, reparability, tendon healing, and functional outcomes, although they are inconsistently applied as formal defining criteria for MRCTs.

Therefore, the aim of this systematic review was to evaluate the role of GTE in the MRI-based definition and structural characterization of MRCTs. Specifically, we sought to determine whether GTE is currently applied as a definitional and quantifiable imaging parameter, how it is assessed across the literature, and how the extent and methodological maturity of the available evidence on GTE relate to those for established MRI-derived variables commonly used in the evaluation of MRCTs. GTE was the primary focus of this review; established MRI-derived parameters served as descriptive context, and no direct comparison of diagnostic accuracy, prognostic performance, or clinical utility between GTE and established parameters was intended.

## Materials and methods

### Protocol and reporting standard

This systematic review was conducted in accordance with the Preferred Reporting Items for Systematic Reviews and Meta-Analyses (PRISMA 2020) statement. The initial search strategy was developed before study screening. Search methods and database-specific strategies were reported in accordance with PRISMA-S. During revision, the GTE-specific search component was expanded to include additional terminology related to footprint exposure and coverage. The review was conceptually focused on MRCTs as a structurally defined entity. Studies addressing irreparability were not excluded a priori; however, irreparability was considered an outcome-related or intraoperative construct rather than a primary imaging-based definition of MRCTs. Accordingly, such studies were included only if they reported preoperative imaging-derived parameters relevant to the structural characterization or assessment of MRCTs. The review was not prospectively registered, and no separate review protocol was published.

### Information sources

A comprehensive electronic literature search was conducted in PubMed/MEDLINE, Embase, and the Cochrane Central Register of Controlled Trials (CENTRAL). All databases were searched from inception to 17 February 2026. To capture additional terminology related to greater tuberosity exposure and footprint coverage, the search was updated on 9 and 11 October 2026 using extended search strings (see Search strategy). In addition, forward and backward citation searching of the Delphi consensus study by Schumaier et al. and of the study by Jo et al. was performed in Scopus. No restrictions regarding study design or language were applied during the electronic search; however, non-English studies were excluded during screening.

### Search strategy

The search strategy consisted of two complementary search strings addressing distinct but related domains of the review question:

1. Greater tuberosity exposure or coverage in the context of rotator cuff tears; and.
2. Imaging-based definitions, structural severity parameters, and measurement properties of MRCTs.

Controlled vocabulary terms, including Medical Subject Headings (MeSH) in PubMed and Emtree terms in Embase, were combined with free-text terms in titles, abstracts, and keywords. The search strategy was adapted to the syntax and indexing system of each database. Partial conceptual overlap between search strings was accepted to maximize sensitivity.

The first search string targeted studies specifically addressing imaging-based evaluation of greater tuberosity exposure (GTE) or footprint coverage. Search terms related to rotator cuff tears were combined with terms referring to the greater tuberosity, including “tuberculum majus,” as well as terms describing “exposure” or “coverage”. For the update search, this string was extended by stand-alone phrases describing footprint exposure or coverage without a mandatory reference to the greater tuberosity (e.g., “bare footprint,” “uncovered footprint,” “exposed greater tuberosity,” “footprint defect,” “residual footprint,” and “tendon coverage”, including singular and plural variants), and the rotator cuff concept was broadened to “rotator cuff”.

The second search string was broader and aimed to identify studies addressing imaging-based definitions, classifications, structural severity indicators, quantitative grading systems, measurement thresholds, reliability, and reproducibility in MRCTs. Studies evaluating associations between imaging findings and irreparability, structural healing, or clinical outcomes were included where applicable.

Because GTE represents a relatively novel and inconsistently reported concept, a broad search strategy was employed to minimize the risk of overlooking studies addressing related footprint-based or structural severity parameters. Complete database-specific search strategies are provided in Supplementary Material 1.

### Inclusion criteria

Studies were eligible if they investigated massive or large-to-massive rotator cuff tears and used MRI or an imaging method with findings transferable to MRI-based structural assessment. In addition, studies had to report at least one of the following:

1. an imaging-based definition or classification of MRCTs;
2. an imaging-derived structural parameter or measurement method; or.
3. an association between a preoperative imaging parameter and reparability, structural healing, or a clinical outcome.

Narrative reviews, expert-opinion papers, or classification proposals were eligible only if they introduced or applied an imaging-based measurement methodology — i.e., defined imaging planes, anatomical landmarks, or quantifiable geometric parameters — for characterizing tear morphology relevant to MRCTs, even where the overall evidence level was expert opinion. Consensus-derived definitions or thresholds proposed without an accompanying imaging-based measurement methodology (e.g., Delphi-derived criteria) did not meet this criterion.

### Exclusion criteria

Studies were excluded if they met any of the following criteria:

1. Not published in English.
2. Full text unavailable.
3. Case reports or single-case studies.
4. Review articles, expert-opinion papers, editorials, or consensus/Delphi statements that proposed definitional criteria or thresholds without an accompanying standardized imaging-based measurement methodology (defined planes, landmarks, or quantification protocol). Consistent with its role as the index publication defining the review question (see Introduction), the Delphi consensus study by Schumaier et al. was not eligible for inclusion in the formal evidence synthesis.
5. Imaging used solely for diagnostic confirmation without structural or quantitative assessment.
6. No relevant imaging-based definition, classification, measurement method, structural parameter, or outcome-related imaging assessment applicable to MRCTs.
7. Exclusive focus on irreparable rotator cuff tears without separate or transferable structural characterization of MRCTs.
8. Cohort definition based solely on intraoperative irreparability without preoperative imaging-based assessment; postoperative or procedure-focused studies were included only if they reported preoperative MRI-derived structural parameters as predictors, and were excluded if their primary focus was surgical technique or postoperative rehabilitation without preoperative structural imaging data.
9. Duplicate or overlapping study populations.
10. Exclusive reliance on non-MRI imaging without transferable relevance to MRI-based assessment.

### Selection process

All retrieved records, including those of the update search and the citation searches, were exported to EndNote 2025 (Clarivate), where duplicates were removed automatically and subsequently by manual comparison of DOI, title, and abstract. Five citation-search records that could not be imported were screened manually on the source platform and were included in the screening counts. Trial registry entries, conference proceedings volumes, and conference abstracts of studies that were also published in full were not screened as separate records. Titles and abstracts and subsequently full texts were screened by one reviewer, and the lists of included and excluded studies were checked by a second reviewer. Data extraction and risk-of-bias assessment were performed by one reviewer. A second reviewer checked the inclusion decisions and the risk-of-bias ratings; the extracted data were not verified in full by a second reviewer. Disagreements were resolved by discussion and consensus. Reports for which the full text could not be retrieved despite reasonable effort were documented as reports not retrieved in the PRISMA flow diagram. Following the clarification of exclusion criterion 8 during revision, all full-text reports previously excluded under this criterion were re-assessed against the revised wording.

### Data extraction and data items

Data extraction included author and year of publication, study design, sample size, definition of massive or large-to-massive rotator cuff tears, imaging modality, MRI-derived structural parameters, measurement methods, anatomical landmarks, reported thresholds, reliability data, investigated outcomes, and any assessment related to GTE or footprint coverage.

Reports describing explicitly duplicate or overlapping study populations were excluded. No additional formal procedure to identify potentially overlapping cohorts was applied; unrecognized overlap between publications from the same institutions or author groups therefore cannot be excluded.

A direct quantitative assessment of GTE was defined as an imaging-based measurement of the exposed proportion of the greater tuberosity or rotator cuff footprint using specified imaging planes, anatomical landmarks, and a reproducible quantification method. Reliability testing and evaluation against structural or clinical outcomes were considered separate indicators of methodological validation.

### Data synthesis

Studies were grouped according to the reported MRCT definition, imaging parameter or measurement approach, and the investigated structural or clinical outcome. Owing to substantial clinical and methodological heterogeneity, findings were synthesized narratively and no quantitative pooling was performed.

### Risk of bias assessment

Study design was classified based on the sampling strategy and temporality described in the methods section of each publication (e.g., whether the full eligible population was enrolled and subsequently stratified by outcome, versus whether cases and controls were specifically selected or matched from a defined source population), independent of the terminology used by the original authors. Methodological quality was assessed using tools appropriate to study design. Prognostic studies evaluating associations between imaging parameters and structural or clinical outcomes were assessed using the Quality In Prognosis Studies (QUIPS) tool. Only studies with a clearly defined prognostic aim were included in this assessment. Domains were rated as low, moderate, or high risk of bias; no overall judgment was derived, and domain-level judgments are reported in Supplementary Table S3.

Prognostic studies evaluating associations between imaging parameters and structural or clinical outcomes were assessed using QUIPS. Other included studies that did not have a prognostic design were appraised narratively with regard to the clarity of the reported definitions, specification of imaging planes and anatomical landmarks, reliability assessment, and clinical applicability. Contextual publications that did not meet the eligibility criteria, including the Schumaier Delphi consensus, were not subjected to formal risk-of-bias assessment.

## Results

### Study selection

**Fig. 1:**
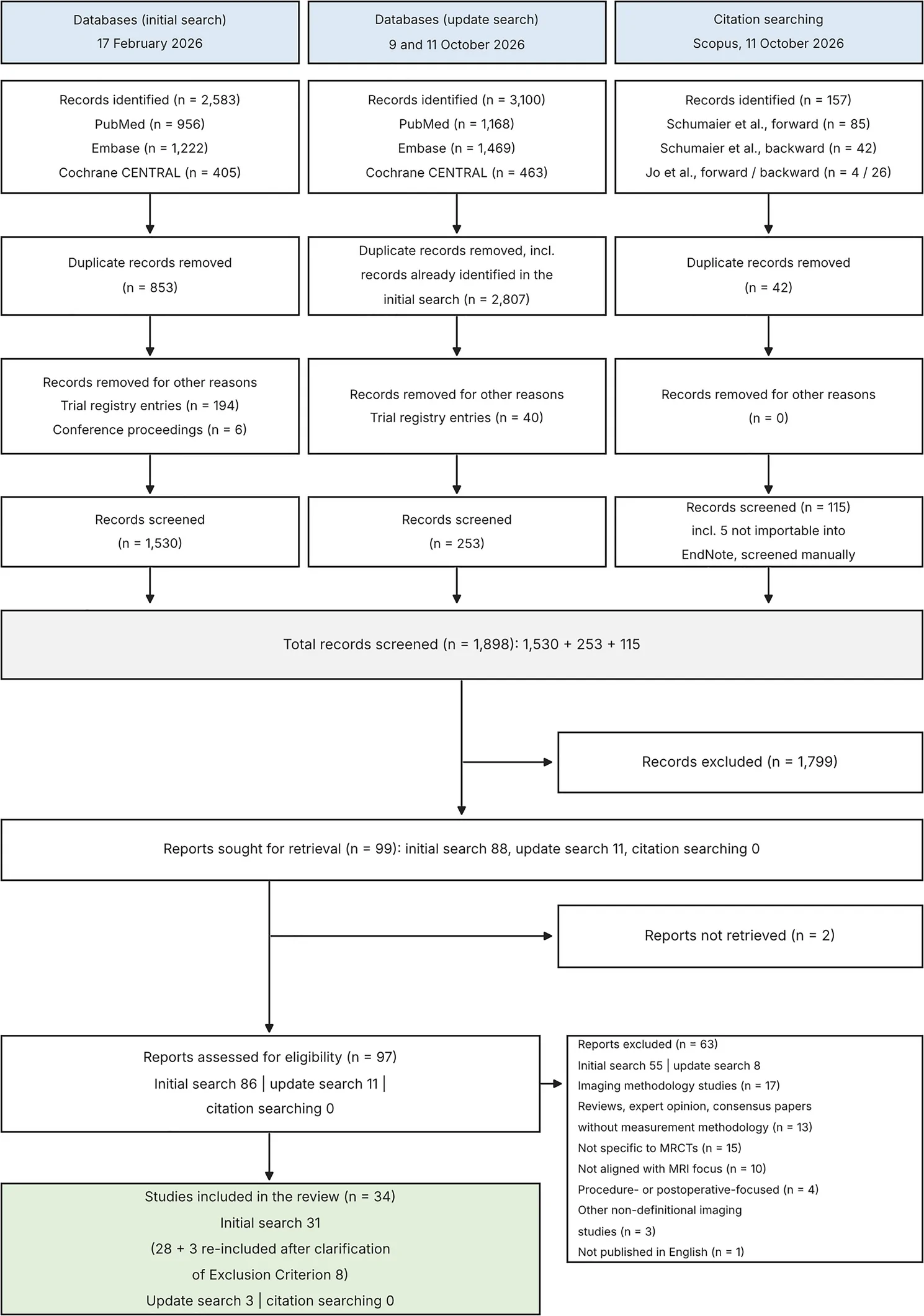
Flow chart illustrating the identification, screening, eligibility assessment, and final inclusion of studies in the narrative synthesis according to PRISMA 2020 guidelines. The diagram follows the PRISMA 2020 template for updated reviews and shows the initial database search (17 February 2026), the update search (9 and 11 October 2026), and citation searching (Scopus, 11 October 2026)

Search results from all databases were merged prior to screening, and the study selection process was documented using a PRISMA 2020 flow diagram (Fig. 1). The initial database search (17 February 2026) identified a total of 2,583 records. Of these, 105 records were retrieved from Embase, 7 from CENTRAL, and 90 from PubMed through Search String 1, while Search String 2 yielded an additional 1,117 records from Embase, 398 from CENTRAL, and 866 from PubMed. After removal of 853 duplicates, 194 trial registry entries, and 6 conference proceedings volumes, 1,530 unique records underwent title and abstract screening. Eighty-eight reports were sought for retrieval. Two reports could not be retrieved, leaving 86 reports for full-text eligibility assessment.

An update search with the extended search strategy (9 and 11 October 2026) identified 3,100 records. After removal of 2,807 records that had already been identified in the initial search or represented duplicates, and 40 trial registry entries, 253 records underwent title and abstract screening. Forward and backward citation searching of Schumaier et al. and Jo et al. in Scopus identified 157 records, of which 42 were duplicates and were removed. The remaining 115 records were screened, including 5 records that could not be imported into EndNote and were screened manually for relevance. In total, 1,898 records were screened across the three search arms. All 11 reports assessed in full text from these two additional arms originated from the update search; no record from citation searching proceeded to full-text assessment.

Of the 97 full-text articles assessed for eligibility (86 from the initial search and 11 from the update search), 63 were excluded for the following reasons: imaging methodology studies without clinically relevant definitional or prognostic analyses (*n* = 17); review articles, expert opinions, or consensus papers without an accompanying imaging-based measurement methodology (*n* = 13); studies not specific to MRCTs (*n* = 15); studies not aligned with the MRI-based focus of the review (*n* = 10); procedure- or postoperative-focused studies (*n* = 4); other non-definitional imaging studies (*n* = 3); and studies not published in English (*n* = 1). Detailed reasons for exclusion of full-text articles are provided in Supplementary Table S1. Following the clarification of exclusion criterion 8, the six reports of the initial search that had been excluded as procedure- or postoperative-focused were re-assessed; three of them reported preoperative MRI-derived structural parameters as predictors in cohorts of massive or large-to-massive tears and were therefore included [7–9]. A total of 34 studies were included in the narrative synthesis (Table 1): 28 from the initial search, three further studies from the initial search after re-assessment [7–9], and three studies from the update search [10–12]. Citation searching did not yield additional eligible studies.

**Table 1:** Overview of included studies summarizing study design and level of evidence (LoE), sample size, type of MRCT definition, imaging modalities, key MRI-derived parameters, primary study outcomes, and evaluation of greater tuberosity exposure (GTE)

Author (Year)	Study Design (LoE)	Sample Size	MRCT Definition Type	Imaging Modality	Key MRI-Derived Parameters	Primary Outcome	GTE Evaluated
Bedeir et al. (2024) [13]	Retrospective cohort (III)	86	Tendon-based	MRI + X-ray	CSA, AI, FI	Functional outcome	No
Ben et al. (2024) [7]	Retrospective cohort (III)	75	Combined structural	MRI + X-ray	Stump signal-intensity ratio, Patte stage, GFDI, AHD	Clinical outcome / graft integrity	No
Chen et al. (2025) [8]	Case series (IV)	45	Geometric/pattern-based	MRI + X-ray	AHI, Patte stage, FI, supraspinatus atrophy	Clinical outcome / retear	No
Cho & Rhee (2009) [14]	Retrospective cohort (III)	169	Size-based	MRI	Tear size, GFDI, tendon integrity	Structural healing	No
Choi et al. (2014) [15]	Case series (IV)	147	Size-based	MRI + arthroscopy	Tear size, Sugaya classification, FI	Clinical and structural outcome	No
Collin et al. (2014) [16]	Cross-sectional study (III)	100	Tendon-based	CT arthrography + X-ray	Tear pattern, FI	Functional outcome / pseudoparalysis	No
Davidson & Burkhart (2010) [4]	Expert opinion (V)	–	Geometric/pattern-based	MRI + X-ray	Tear geometry, FI	Classification concept	No
Davidson et al. (2005) [5]	Retrospective cohort (III)	66 (55 analyzed)	Geometric/pattern-based	MRI	Tear size (L/W)	Structural configuration	No
Di Benedetto et al. (2019) [17]	Retrospective cohort (III)	131	Combined structural	MRI	FI, Patte stage, tear size, AHD	Reparability	No
Dwyer et al. (2015) [18]	Retrospective cohort (III)	60	Combined structural	MRI	Tear size, Patte stage, FI, muscle atrophy, AHD	Reparability	No
Ernstbrunner et al. (2020) [19]	Case-control study (III)	50	Tendon-based	MR arthrography + X-ray	Tear extension, FI, CSA, AHD	Functional outcome	No
Fonseca et al. (2025) [20]	Cross-sectional study (III)	71 shoulders	Tendon-based	MRI + X-ray	Tear extension, FI, muscle atrophy, Patte stage	Functional outcome	No
Furuhata et al. (2022) [21]	Retrospective cohort study (III)	204	Tendon-based	MRI + X-ray	Tendon pattern, FI, AHI	Range of motion	No
Gallego et al. (2026) [10]	Retrospective cohort (III)	57	Combined structural	MRI + X-ray	Tear size, tangent sign, FI	Clinical outcome / revision	No
Gerber et al. (2000) [3]	Prospective case series (IV)	29 (27 follow-up)	Tendon-based	MRI + X-ray	Muscle cross-sectional area, FI, AHD	Clinical and structural outcome	No
Guo et al. (2020) [22]	Retrospective cohort study (III)	121 shoulders	Combined structural	MRI	Patte stage, FI, AHD, tangent sign	Reparability and retear	No
Guo et al. (2023) [23]	Retrospective cohort study (III)	187	Size-based	MRI	FI, Patte stage, occupation ratio, AHD	Functional outcome	No
Iijima et al. (2019) [24]	Prospective cohort (II)	107	Size-based	MRI	T2 mapping, FI, Sugaya classification	Structural healing / retear	No
Itoigawa et al. (2026) [11]	Retrospective cohort (III)	102	Size-based	MRI + ultrasound (SWE)	Tear size, FI	Reparability	No
Jo et al. (2025) [25]	Case series (IV)	57	Footprint-based	MRI	GT coverage, FI, nCSA, Sugaya classification	Clinical and structural outcome	Qualitative only
Kim J.Y. et al. (2017) [26]	Retrospective cohort study (III)	105	Tendon-based	MRI	FI, Patte stage, tangent sign	Reparability	No
Kim S.J. et al. (2016) [27]	Retrospective cohort (III)	87	Combined structural	MRI	Tear size, Patte stage, FI, IGHD	Reparability	No
Kuptniratsaikul et al. (2019) [28]	Retrospective cohort (III)	66	Combined structural	MRI + X-ray	Tear size, AHI, FI, Patte stage	Reparability	No
Mellado et al. (2005) [29]	Retrospective cohort (III)	28	Combined Structural	MRI	Tendon integrity, FI, muscle cross-sectional area	Clinical outcome	No
Ohzono et al. (2017) [30]	Retrospective cohort study (III)	155 (55 analyzed)	Combined structural	MRI	FI, tear size, occupation ratio	Clinical outcome	No
Ok et al. (2017) [31]	Prospective cohort (II)	104	Tendon-based	MRI + X-ray	Tear pattern, FI, tendon retraction, AHD	Functional outcome	No
Park et al. (2020) [32]	Retrospective cohort (III)	64	Tendon-based	MRI + X-ray	AHI, IGHD, UMI, FI, Patte stage	Reparability	No
Sheean et al. (2018) [33]	Case series (IV)	86	Combined structural	MRI + X-ray	AHI, FI, tangent sign	Reparability	No
Tanaka et al. (2021) [9]	Case series (IV)	39 (of 196)	Size-based	MRI	GFDI, tear size, semi-quantitative five-zone score of residual tendon attachment	Clinical and structural outcome	Indirect only
Wieser et al. (2015) [34]	Prospective cohort (II)	20 shoulders	Tendon-based	MRI + fluoroscopy	FI, tear extension, CSA, AHD	Functional outcome	No
Xie et al. (2025) [35]	Retrospective cohort (III)	105	Size-based	MRI	Fat percentage, FI, Patte stage, AHD	Structural healing / retear	No
Ying et al. (2025) [36]	Retrospective cohort (III)	394 shoulders	Geometric/pattern-based	MRI + arthroscopy	Tear pattern, healing integrity	Prognostic outcome	No
Yoo et al. (2009) [37]	Prospective case series (IV)	51	Size-based	MRI or MR arthrography	FI, tendon retraction (COTD/SOTD)	Reparability	No
Yoshimura et al. (2026) [12]	Retrospective cohort (III)	177 shoulders	Size-based	MRI	Shift angle, medial retraction, tear size, FI, tangent sign, AHI	Reparability	No

**Abbreviations**: AHD, acromiohumeral distance; AHI, acromiohumeral interval; AI, acromial index; COTD, coronal-oblique tendon displacement; CSA, critical shoulder angle; CT, computed tomography; FI, fatty infiltration; GFDI, global fatty degeneration index; GT, greater tuberosity; GTE, greater tuberosity exposure; IGHD, inferior glenohumeral distance; L/W, length/width; MR, magnetic resonance; MRI, magnetic resonance imaging; MRCT, massive rotator cuff tear; nCSA, normalized critical shoulder angle; SOTD, sagittal-oblique tendon displacement; SWE, shear wave elastography; UMI, upward migration index. “Indirect only”: footprint attachment assessed by a semi-quantitative score rather than GTE

### Risk of bias

#### Prognostic studies (QUIPS)

**Fig. 2:**
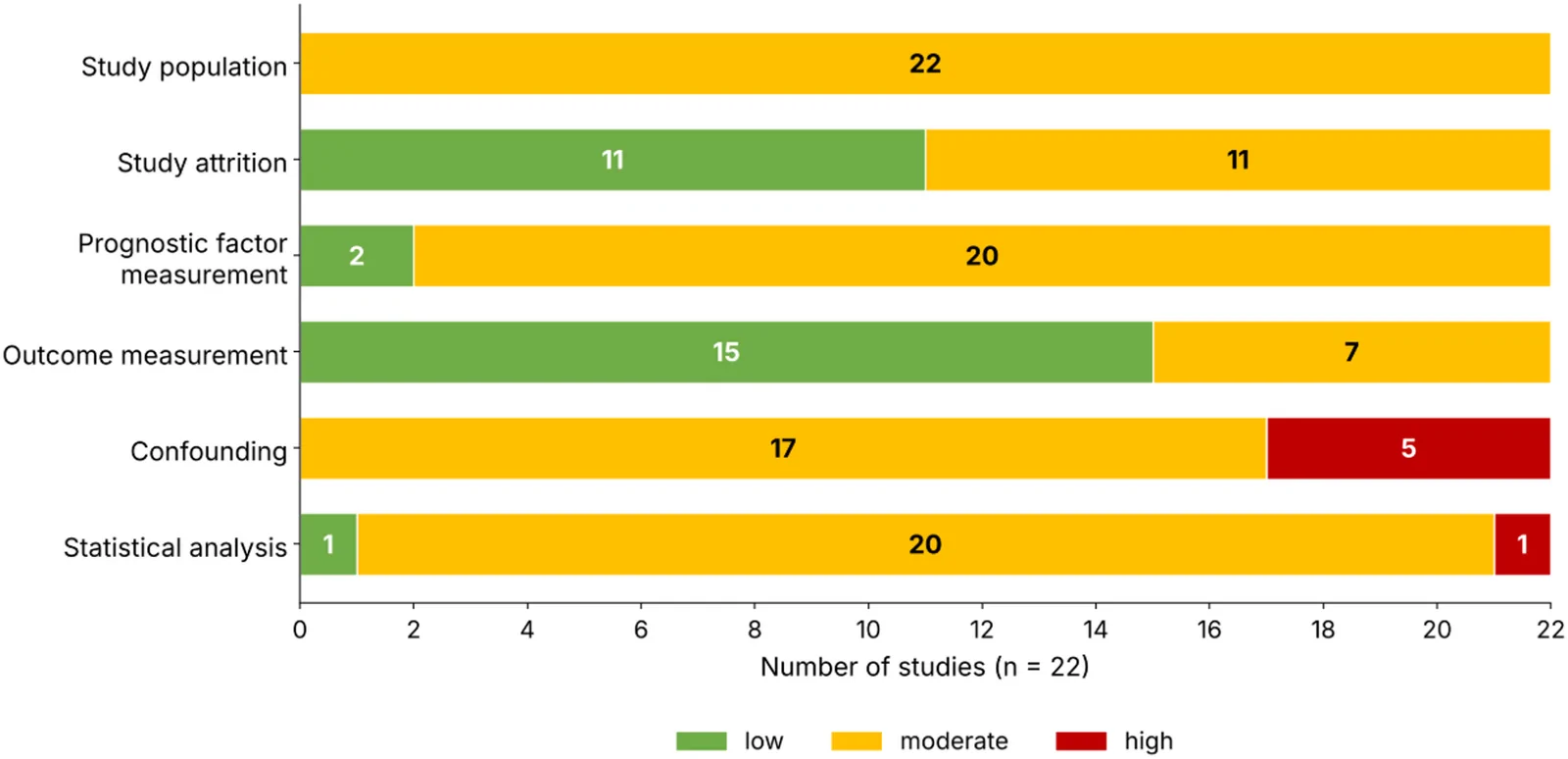
Risk-of-bias assessment of included prognostic studies (*n* = 22) using the Quality In Prognosis Studies (QUIPS) tool. Each domain was rated as low (green), moderate (yellow), or high (red) risk of bias; no overall judgment was derived. Confounding represented the most frequent source of high risk of bias (5 of 22 studies), whereas outcome measurement was generally associated with low risk of bias across studies (15 of 22 studies)

A total of 22 studies with a prognostic design were assessed using the QUIPS tool (Fig. 2). Individual studies and their domain-level judgments are listed in Supplementary Table S3. Risk-of-bias judgments varied across domains: study participation was rated moderate in all 22 studies and outcome measurement low in 15, whereas the most frequent concerns related to confounding (high risk in 5 studies) and, at moderate risk, to statistical analysis and prognostic factor measurement (20 studies each). No overall QUIPS judgment was derived; domain-level judgments are provided in Supplementary Table S3. Most studies were retrospective cohort or case-control studies. The principal source of bias was inadequate control of confounding variables, including patient-related and treatment-related factors, resulting in moderate to high risk of bias in this domain.

Outcome assessment was generally robust when based on objective structural endpoints, particularly MRI-defined tendon healing or retear. Prognostic factor measurement demonstrated moderate risk of bias due to heterogeneity in imaging protocols, measurement techniques, and parameter definitions. Nevertheless, commonly used MRI-derived parameters—such as fatty infiltration, tendon retraction, tear size, and acromiohumeral distance—were typically assessed using established methodologies.

Overall, risk of bias was driven primarily by confounding and methodological heterogeneity rather than study design alone.

#### Non-prognostic and conceptual studies

Studies not suitable for QUIPS assessment were evaluated narratively. Across these studies, the main limitation was the lack of standardization in imaging acquisition and measurement protocols, with substantial heterogeneity in imaging planes, measurement techniques, and applied thresholds.

Conceptual and descriptive studies further demonstrated variability in definitions and classification approaches, frequently combining imaging findings with intraoperative or clinical criteria without consistent standardization.

### Overall interpretation

Overall, the available evidence is characterized by moderate methodological limitations, predominantly related to retrospective study designs, inadequate control of confounding variables, and substantial heterogeneity in imaging protocols and assessment strategies.

Importantly, established MRI-derived parameters are generally evaluated using established measurement frameworks and are frequently evaluated in relation to structural severity, reparability, tendon healing, and functional outcomes, notwithstanding methodological heterogeneity across studies. In contrast, GTE is neither systematically assessed nor validated as a reproducible quantitative imaging parameter within the current literature. This contrast concerns the extent and methodological maturity of the available evidence and does not represent a comparison of the reliability, diagnostic accuracy, or prognostic performance of GTE and established parameters.

### Definitions of massive rotator cuff tears

Definitions of MRCTs were highly heterogeneous and lacked a standardized imaging-based framework. Across all included studies, MRCTs were defined using indirect structural parameters, whereas direct measures of footprint deficiency, such as greater tuberosity exposure (GTE), were neither applied as formal definitional criteria nor quantitatively assessed.

Two principal definitional approaches predominated in the literature. The variability in definitional strategies across included studies is summarized in Table 2 and highlights the absence of a consistent imaging-based framework. Most studies defined MRCTs based on tendon involvement, typically requiring complete detachment of two or more rotator cuff tendons [3, 16, 19, 21, 31, 34]. Alternatively, size-based definitions were used, most commonly applying thresholds greater than 5 cm or at least 3 cm in the context of large-to-massive tears [9, 11, 12, 14, 15, 24, 35, 37]. Table 2 summarizes the 24 included studies for which an explicit or clearly derivable imaging-based MRCT definition was reported.

**Table 2:** Summary of MRI-based definitions of massive rotator cuff tears (MRCTs) in 24 of the 34 included studies that reported an explicit or clearly derivable imaging-based definition

Author (Year)	Definition Type	MRCT Definition (Imaging-Based)	Key Concept	GTE Included
Ben et al. (2024) [7]	Combined structural	Greatest tear size > 5 cm, complete tear of ≥ 2 tendons, or Patte grade ≥ 3 retraction	Tear size, tendon involvement, and retraction	No
Chen et al. (2025) [8]	Geometric/pattern-based	Anterior L-shaped tear > 5 cm (AP) involving ≥ 2 tendons with extension into the rotator interval	Tear geometry and tendon involvement	No
Cho & Rhee (2009) [14]	Size-based	Tear size > 5 cm	Tear size	No
Choi et al. (2014) [15]	Size-based	Tear size > 5 cm	Tear size	No
Collin et al. (2014) [16]	Tendon-based	≥ 2 tendons involved with advanced fatty infiltration	Tendon involvement and muscle degeneration	No
Davidson & Burkhart (2010) [4]	Geometric/pattern-based	Geometric tear classification (length/width patterns)	Tear geometry	No
Davidson et al. (2005) [5]	Geometric/pattern-based	Bidimensional tear geometry	Tear configuration	No
Di Benedetto et al. (2019) [17]	Combined structural	Tear size with structural degeneration markers	Tear size, fatty infiltration, and tendon retraction	No
Dwyer et al. (2015) [18]	Combined structural	Large-to-massive tears with structural degeneration	Tear size and degeneration	No
Ernstbrunner et al. (2020) [19]	Tendon-based	≥ 2 tendons detached	Tendon involvement	No
Furuhata et al. (2022) [21]	Tendon-based	≥ 2 tendons involved	Tendon involvement	No
Gallego et al. (2026) [10]	Combined structural	Tear > 5 cm in any dimension or involvement of ≥ 2 posterosuperior tendons	Tear size and tendon involvement	No
Gerber et al. (2000) [3]	Tendon-based	Complete detachment of ≥ 2 tendons	Tendon involvement	No
Iijima et al. (2019) [24]	Size-based	Large tears > 3 cm; massive tears > 5 cm	Tear size	No
Itoigawa et al. (2026) [11]	Size-based	Large or massive tears (> 3 cm)	Tear size	No
Kim et al. (2016) [27]	Combined structural	Tear size > 3 cm combined with structural imaging parameters	Tear size and structural degeneration	No
Kuptniratsaikul et al. (2019) [28]	Combined structural	Large-to-massive tears with MRI-based predictors	Tear size and degeneration	No
Ok et al. (2017) [31]	Tendon-based	≥ 2 tendons with subgroup classification	Tendon involvement pattern	No
Tanaka et al. (2021) [9]	Size-based	Large-to-massive tears (size-based; no threshold specified)	Tear size; indirect footprint attachment score	Indirect only
Wieser et al. (2015) [34]	Tendon-based	≥ 2 complete tendon tears	Tendon involvement	No
Xie et al. (2025) [35]	Size-based	Tear size > 3 cm in large-to-massive tears	Tear size	No
Ying et al. (2025) [36]	Geometric/pattern-based	Geometric MRCT classification based on tear pattern	Tear pattern	No
Yoo et al. (2009) [37]	Size-based	Tear size > 3 cm	Tear size	No
Yoshimura et al. (2026) [12]	Size-based	Large-to-massive tears (≥ 3 cm in AP or ML dimension)	Tear size	No

The table presents the definition type, underlying structural imaging concepts, and consideration of greater tuberosity exposure (GTE). Abbreviations: AP, anteroposterior; GT, greater tuberosity; ML, mediolateral; MRI, magnetic resonance imaging

Several studies combined these approaches by integrating tear size and tendon involvement to describe structural extent [7, 10, 17, 18, 27–29].

Less frequently, geometric or pattern-based definitions were proposed; however, these approaches were inconsistently applied and were not systematically associated with structural or clinical outcomes [4, 5, 8, 36].

Imaging-derived parameters—including tear size, tendon retraction, fatty infiltration, and muscle atrophy—were frequently evaluated in studies involving MRCTs. Importantly, these variables were not used as formal defining criteria but rather as markers of structural severity and predictors of surgical or clinical outcomes [7–12, 18, 26, 33, 35].

None of the included studies implemented GTE as a quantitative imaging-based definition. Only two studies addressed footprint coverage on imaging: one assessed it qualitatively, without predefined thresholds or a standardized measurement approach [25], and one scored the residual tendon attachment of the footprint after repair semi-quantitatively in five zones on MRI [9], which involved neither a direct quantitative measurement of GTE nor a validated GTE threshold.

### Measurement of MRI-derived parameters

No standardized imaging methodology for the assessment of GTE was identified. None of the included studies defined imaging planes, slice selection, anatomical landmarks, or reproducible thresholds for its measurement. Consequently, no standardized and outcome-validated MRI-based method for quantifying the proposed ≥ 67% cutoff was identified within the scope of this review.

In contrast, MRI-derived parameters commonly used in studies of MRCTs—such as tear size, tendon retraction, fatty infiltration, muscle atrophy, and superior humeral migration—were evaluated using established measurement frameworks. Across studies, these parameters were assessed on defined MRI planes and anatomical landmarks and were often associated with clinically interpretable thresholds, although imaging protocols and cutoff values varied between studies [5, 7–12, 17, 18, 22, 26, 28, 30, 32, 35, 37]. The studies added after the amendment of Exclusion Criterion 8 and the update search contributed signal-intensity-based classification of the tendon stump [7], a semi-quantitative five-zone score of residual tendon attachment [9], and deep-layer parameters such as shift angle and medial retraction [12]; inter-observer reliability was reported for four of these studies [7, 9, 11, 12]. An overview of MRI-derived parameters and their measurement characteristics is provided in Supplementary Table S2, illustrating the difference in the extent and methodological maturity of the available evidence between established imaging parameters and GTE, for which no reproducible methodology was identified.

### MRI-based associations with reparability and clinical outcomes

Greater tuberosity exposure (GTE) was not evaluated in relation to irreparability, structural healing, or functional outcomes in any included study. In contrast, reparability outcomes—typically operationalized as the ability to achieve complete footprint coverage at surgery—were frequently evaluated in relation to progressive structural degeneration reflected by established MRI-derived parameters [11, 12, 17, 18, 22, 26–28, 37]. In large-to-massive tears, two further studies related preoperative MRI parameters to intraoperative reparability: medial retraction of the deep layer and supraspinatus fatty infiltration were independently associated with reparability, with shift angle and medial retraction introduced as new deep-layer parameters [12], whereas tear size, but not Goutallier stage, remained associated with irreparability alongside ultrasound shear wave elastography, which was the strongest independent predictor [11].

Structural healing was similarly associated with established imaging-derived variables, particularly tear size, fatty degeneration, and muscle quality [3, 7–9, 14, 29, 35]. Furthermore, a low preoperative signal-intensity ratio of the tendon stump (type 1) was associated with a lower graft failure rate after superior capsular reconstruction [7], a preoperative acromiohumeral interval below 5 mm was the only variable independently associated with retear after partial repair with rotator interval shift [8], and, in patients with retear, a higher preoperative global fatty degeneration index correlated with a smaller residual tendon attachment area [9].

Functional outcomes were related to tear configuration, muscle involvement, and superior humeral migration [7, 9, 10, 13, 16, 19, 20, 34]. In particular, a low preoperative stump signal-intensity ratio was associated with higher American Shoulder and Elbow Surgeons (ASES) and Constant scores after superior capsular reconstruction [7]; in a retrospective cohort of MRCTs, preoperative tear size and infraspinatus fatty infiltration were associated with the Constant score in univariate analysis, but only revision surgery remained independently associated in the multivariable model [10]; and after retear, the preoperative global fatty degeneration index correlated negatively with 24-month Japanese Orthopaedic Association (JOA) and University of California, Los Angeles (UCLA) shoulder scores [9]. Across studies, functional impairment was consistently described as a multifactorial construct reflecting the combined effects of structural damage, tendon involvement, and muscle degeneration.

Indirect references to footprint coverage were limited to one qualitative description that lacked predefined or standardized thresholds [25] and one semi-quantitative five-zone score of residual tendon attachment after repair, which involved neither a direct quantitative measurement of GTE nor a validated GTE threshold [9].

## Discussion

The primary aim of this systematic review was to evaluate the role of greater tuberosity exposure (GTE) in imaging-based definitions and structural assessment of massive rotator cuff tears (MRCTs). The principal finding is that GTE, despite its inclusion in a recent Delphi consensus, has not been operationalized as a direct quantitative MRI-based parameter [6]. None of the included studies measured the exposed proportion of the greater tuberosity using predefined imaging planes, anatomical landmarks, and a reproducible quantification method. Two studies reported related but distinct footprint assessments. Jo et al. qualitatively assessed lateral tendon excursion and greater tuberosity coverage in the context of surgical repair [25], whereas Tanaka et al. used a semiquantitative five-zone score to assess residual tendon attachment before and after repair in a selected cohort of patients with postoperative retears [9]. Neither approach quantified GTE as an exposure parameter or evaluated the proposed ≥ 67% threshold. This threshold therefore remains consensus-derived rather than an operational and validated imaging criterion. Of the 63 reports excluded after full-text assessment, 17 focused on imaging methods for established parameters, including fatty infiltration, tear morphology, and acromiohumeral distance. None described a direct quantitative GTE measurement, supporting the conclusion that such a method was not identified within the scope of this review. The evidence available for established MRI-derived parameters was substantially more extensive and methodologically mature. Tear size, tendon involvement, tendon retraction, fatty infiltration, muscle atrophy, and superior humeral migration were assessed using defined imaging planes, anatomical landmarks, or established classification systems, although imaging protocols and thresholds varied across studies [5, 11, 12, 17, 18, 22, 27, 28, 35, 37]. These parameters were frequently investigated in relation to reparability, structural healing, and functional outcomes [3, 7, 9, 10, 14, 16, 19, 31, 34, 35], and progressive structural degeneration was commonly associated with a reduced likelihood of complete repair [11, 12, 17, 18, 22, 26–28, 37]. This contrast reflects differences in the extent and methodological maturity of the available evidence. It does not represent a direct comparison of reliability, diagnostic accuracy, prognostic performance, feasibility, or clinical utility. Accordingly, neither clinical inferiority of GTE nor superiority of the established parameters can be inferred. The absence of a validated GTE method should be interpreted as an unresolved methodological gap rather than evidence that the concept lacks clinical value.

A further finding was the persistent heterogeneity in definitions of MRCTs. Most studies classified tears according to tendon involvement, tear size, or geometric configuration [3–5, 14, 16, 31, 36]. Irreparability, in contrast, was generally treated as a distinct operative or outcome-related construct reflecting the inability to achieve adequate tendon mobilization and complete anatomical repair, rather than as a defining feature of an MRCT [18, 26, 33]. Although the two concepts are closely related, they are not interchangeable: MRCT describes the structural extent or configuration of a tear, whereas irreparability depends additionally on tendon mobility, tissue quality, chronic degeneration, and the operative criteria applied. Failure to distinguish these concepts contributes to heterogeneity across studies and complicates the integration of emerging parameters such as GTE into a consistent imaging-based framework.

### Limitations

This review has several limitations. First, the included studies were heterogeneous with respect to study design, imaging protocols, and outcome definitions, limiting direct comparability across studies. Most prognostic studies were retrospective and demonstrated moderate methodological limitations, particularly regarding control of confounding variables. Second, irreparability was frequently defined intraoperatively, introducing subjectivity and limiting reproducibility. In addition, definitions of both “massive” and “irreparable” rotator cuff tears remain inconsistent, further complicating cross-study comparisons. Third, because the conclusions regarding GTE rest on an absence of evidence, this finding should not be interpreted as evidence that GTE lacks clinical value, but rather as an unresolved methodological gap warranting dedicated validation studies. Fourth, title and abstract screening, full-text assessment, and data extraction were performed by a single reviewer; a second reviewer checked the lists of included and excluded studies, the inclusion decisions, and the risk-of-bias ratings, but the extracted data were not independently verified in full. The lack of independent duplicate screening and of duplicate data extraction may have introduced selection or extraction bias. In addition, no additional formal procedure to identify potentially overlapping cohorts was applied, so unrecognized overlap between publications from the same institutions or author groups cannot be excluded.

Furthermore, eligibility criteria were broad and involved some subjective judgment, particularly regarding the classification of studies as clinically relevant; imaging-methodology studies without accompanying clinical or definitional analysis were excluded, which may have limited the identification of GTE-relevant measurement approaches. Formal risk-of-bias assessment was not performed for descriptive, conceptual, and consensus-based studies. Combining formally assessed (QUIPS) and narratively appraised evidence within a single synthesis introduces heterogeneity in the rigor of quality assessment across included studies, and findings drawn from narratively appraised studies should be interpreted with corresponding caution.

Finally, although the updated search incorporated controlled vocabulary, additional terminology related to footprint exposure and coverage, and supplementary citation searching, the strategy was not formally peer-reviewed by an information specialist. The possibility that studies using unanticipated terminology were missed therefore cannot be entirely excluded. Moreover, the juxtaposition of GTE and established MRI-derived parameters was limited to the extent and methodological maturity of the available evidence; because GTE was not quantitatively evaluated in any included study, no conclusions regarding comparative reliability, diagnostic accuracy, or prognostic performance can be drawn.

Despite these limitations, this review provides a structured synthesis of MRI-derived parameters used in the assessment of MRCTs and highlights a critical gap between emerging conceptual definitions and the currently available empirical evidence.

## Conclusion

GTE has recently emerged as a proposed imaging-based criterion for the definition of massive rotator cuff tears. However, it currently remains a conceptual construct without standardized measurement protocols or evidence linking it to clinically relevant structural or functional outcomes. The evidence base for established MRI-derived parameters—including tendon retraction, fatty infiltration, muscle atrophy, and tear size—is more extensive and methodologically mature, as these parameters are embedded in established measurement frameworks and are frequently evaluated, albeit heterogeneously, in relation to structural severity, reparability, tendon healing, and clinical outcomes. Future research should focus on the development and validation of standardized MRI-based measurement techniques for GTE and evaluate its diagnostic and prognostic relevance. At present, this absence of standardization and outcome-based validation—rather than evidence against its clinical value—distinguishes the current evidence on GTE from that on established MRI-derived parameters. This comparison of the maturity of the available evidence does not imply clinical inferiority of GTE or superiority of established parameters.

## Supplementary Information

Below is the link to the electronic supplementary material.

Supplementary Material 1

## Data Availability

All data extracted or generated during this systematic review are included in this article and its supplementary information files.
