# Supplementary material for: MRI-based assessment of greater tuberosity exposure in massive rotator cuff tears: a systematic review of current evidence and methodological gaps
Source: BMC Musculoskelet Disord. 2026 Nov 3;27:971. doi: 10.1186/s12891-026-10705-3 (PMC13753500; doi:10.1186/s12891-026-10705-3)
Supplement: Supplementary file 1 [file 12891_2026_10705_MOESM1_ESM.docx]

**Supplementary Data**

*Database 1: PubMed (MEDLINE) – initial search (17 February 2026)*

**Platform:** NCBI

**Coverage:** From inception to 17 February 2026

**Date of last search:** 17 February 2026

**Limits applied:** None

**Results:** Search 1 = 90; Search 2 = 866

**Search Strategy 1 – Greater Tuberosity Exposure/Coverage**

(("Rotator Cuff Injuries"[Mesh] OR "rotator cuff tear*"[tiab] OR "rotator cuff rupture*"[tiab]) AND ("greater tuberosity"[tiab] OR "tuberculum majus"[tiab]) AND (exposure[tiab] OR coverage[tiab] OR uncover*[tiab] OR footprint[tiab]))

**Search Strategy 2 – Imaging-Based Definitions and Structural Parameters**

(("Rotator Cuff Injuries"[Mesh] OR "rotator cuff tear*"[tiab] OR "rotator cuff rupture*"[tiab]) AND (massive[tiab] OR irreparable[tiab] OR repairability[tiab] OR reparable[tiab]) AND ("Diagnostic Imaging"[Mesh] OR "Magnetic Resonance Imaging"[Mesh] OR imaging[tiab] OR radiolog*[tiab] OR MRI[tiab] OR "magnetic resonance"[tiab]) AND (criteria[tiab] OR definition*[tiab] OR classification*[tiab] OR parameter*[tiab] OR predictor*[tiab] OR grading[tiab] OR measurement[tiab] OR quantitat*[tiab] OR reliability[tiab] OR reproducibility[tiab] OR interobserver[tiab] OR intraobserver[tiab] OR cutoff[tiab] OR threshold[tiab] OR Goutallier[tiab] OR Patte[tiab] OR "fatty infiltration"[tiab] OR retraction[tiab] OR "muscle atrophy"[tiab] OR "tangent sign"[tiab] OR "occupation ratio"[tiab] OR "acromiohumeral distance"[tiab] OR "number of tendons"[tiab] OR "tendon involvement"[tiab] OR outcome*[tiab] OR function*[tiab] OR prognosis[tiab] OR healing[tiab] OR retear[tiab] OR postoperative[tiab]))

*Database 1 (update search): PubMed (MEDLINE)*

**Platform:** NCBI

**Coverage:** From inception to 9 October 2026 (update search; initial search: inception to 17 February 2026)

**Date of last search:** 9 October 2026

**Limits applied:** None

**Results:** Search 1 = 216; Search 2 = 952

**Search Strategy 1 – Greater Tuberosity Exposure/Coverage (extended)**

(("Rotator Cuff Injuries"[Mesh] OR "rotator cuff"[tiab]) AND ((("greater tuberosity"[tiab] OR "tuberculum majus"[tiab]) AND (exposure[tiab] OR coverage[tiab] OR uncover*[tiab] OR footprint*[tiab])) OR "greater tuberosity exposure"[tiab] OR "tuberosity exposure"[tiab] OR "footprint exposure"[tiab] OR "exposed footprint"[tiab] OR "exposed footprints"[tiab] OR "uncovered footprint"[tiab] OR "uncovered footprints"[tiab] OR "bare footprint"[tiab] OR "bare footprints"[tiab] OR "bare tuberosity"[tiab] OR "bare tuberosities"[tiab] OR "tendon coverage"[tiab] OR "footprint coverage"[tiab] OR "residual footprint"[tiab] OR "residual footprints"[tiab] OR "footprint defect"[tiab] OR "footprint defects"[tiab] OR "uncovered tuberosity"[tiab] OR "uncovered tuberosities"[tiab] OR "uncovered greater tuberosity"[tiab] OR "exposed greater tuberosity"[tiab]))

**Search Strategy 2 – Imaging-Based Definitions and Structural Parameters (extended)**

(("Rotator Cuff Injuries"[Mesh] OR "rotator cuff tear*"[tiab] OR "rotator cuff rupture*"[tiab]) AND (massive[tiab] OR irreparable[tiab] OR repairability[tiab] OR reparable[tiab]) AND ("Diagnostic Imaging"[Mesh] OR "Magnetic Resonance Imaging"[Mesh] OR imaging[tiab] OR radiolog*[tiab] OR MRI[tiab] OR "magnetic resonance"[tiab]) AND ( criteria[tiab] OR definition*[tiab] OR classification*[tiab] OR parameter*[tiab] OR predictor*[tiab] OR grading[tiab] OR measurement[tiab] OR quantitat*[tiab] OR reliability[tiab] OR reproducibility[tiab] OR interobserver[tiab] OR intraobserver[tiab] OR cutoff[tiab] OR threshold[tiab] OR Goutallier[tiab] OR Patte[tiab] OR "fatty infiltration"[tiab] OR retraction[tiab] OR "muscle atrophy"[tiab] OR "tangent sign"[tiab] OR "occupation ratio"[tiab] OR "acromiohumeral distance"[tiab] OR "number of tendons"[tiab] OR "tendon involvement"[tiab] OR outcome*[tiab] OR function*[tiab] OR prognosis[tiab] OR healing[tiab] OR retear[tiab] OR postoperative[tiab] OR footprint*[tiab] OR "greater tuberosity exposure"[tiab] OR "tuberosity exposure"[tiab] OR "footprint exposure"[tiab] OR "exposed footprint"[tiab] OR "exposed footprints"[tiab] OR "uncovered footprint"[tiab] OR "uncovered footprints"[tiab] OR "bare footprint"[tiab] OR "bare footprints"[tiab] OR "bare tuberosity"[tiab] OR "bare tuberosities"[tiab] OR "tendon coverage"[tiab] OR "footprint coverage"[tiab] OR "residual footprint"[tiab] OR "residual footprints"[tiab] OR "footprint defect"[tiab] OR "footprint defects"[tiab] OR "uncovered tuberosity"[tiab] OR "uncovered tuberosities"[tiab] OR "uncovered greater tuberosity"[tiab] OR "exposed greater tuberosity"[tiab]))

*Database 2: Embase – initial search (17 February 2026)*

**Platform:** Elsevier

**Coverage:** From inception to 17 February 2026

**Date of last search:** 17 February 2026

**Limits applied:** None

**Results:** Search 1 = 105; Search 2 = 1117

**Search Strategy 1 – Greater Tuberosity Exposure/Coverage**

('rotator cuff injury'/exp OR 'rotator cuff tear':ti,ab,kw) AND ('greater tuberosity':ti,ab,kw OR 'tuberculum majus':ti,ab,kw) AND (exposure:ti,ab,kw OR coverage:ti,ab,kw OR uncover*:ti,ab,kw OR footprint:ti,ab,kw)

**Search Strategy 2 – Imaging-Based Definitions and Structural Parameters**

('rotator cuff injury'/exp OR 'rotator cuff tear':ti,ab,kw OR 'rotator cuff rupture':ti,ab,kw) AND (massive:ti,ab,kw OR irreparable:ti,ab,kw OR repairability:ti,ab,kw OR reparable:ti,ab,kw) AND ('diagnostic imaging'/exp OR 'magnetic resonance imaging'/exp OR imaging:ti,ab,kw OR radiolog*:ti,ab,kw OR mri:ti,ab,kw OR 'magnetic resonance':ti,ab,kw) AND (criteria:ti,ab,kw OR definition*:ti,ab,kw OR classification*:ti,ab,kw OR parameter*:ti,ab,kw OR predictor*:ti,ab,kw OR grading:ti,ab,kw OR measurement:ti,ab,kw OR quantitat*:ti,ab,kw OR reliability:ti,ab,kw OR reproducibility:ti,ab,kw OR interobserver:ti,ab,kw OR intraobserver:ti,ab,kw OR cutoff:ti,ab,kw OR threshold:ti,ab,kw OR goutallier:ti,ab,kw OR patte:ti,ab,kw OR 'fatty infiltration':ti,ab,kw OR retraction:ti,ab,kw OR 'muscle atrophy':ti,ab,kw OR 'tangent sign':ti,ab,kw OR 'occupation ratio':ti,ab,kw OR 'acromiohumeral distance':ti,ab,kw OR 'number of tendons':ti,ab,kw OR 'tendon involvement':ti,ab,kw OR outcome*:ti,ab,kw OR function*:ti,ab,kw OR prognosis:ti,ab,kw OR healing:ti,ab,kw OR retear:ti,ab,kw OR postoperative:ti,ab,kw)

*Database 2 (update search): Embase*

**Platform:** Elsevier

**Coverage:** From inception to 11 October 2026 (update search; initial search: inception to 17 February 2026)

**Date of last search:** 11 October 2026

**Limits applied:** None

**Results:** Search 1 = 245; Search 2 = 1224

**Search Strategy 1 – Greater Tuberosity Exposure/Coverage (extended)**

('rotator cuff injury'/exp OR 'rotator cuff':ti,ab,kw) AND ((('greater tuberosity':ti,ab,kw OR 'tuberculum majus':ti,ab,kw) AND (exposure:ti,ab,kw OR coverage:ti,ab,kw OR uncover*:ti,ab,kw OR footprint*:ti,ab,kw)) OR 'greater tuberosity exposure':ti,ab,kw OR 'tuberosity exposure':ti,ab,kw OR 'footprint exposure':ti,ab,kw OR 'exposed footprint':ti,ab,kw OR 'exposed footprints':ti,ab,kw OR 'uncovered footprint':ti,ab,kw OR 'uncovered footprints':ti,ab,kw OR 'bare footprint':ti,ab,kw OR 'bare footprints':ti,ab,kw OR 'bare tuberosity':ti,ab,kw OR 'bare tuberosities':ti,ab,kw OR 'tendon coverage':ti,ab,kw OR 'footprint coverage':ti,ab,kw OR 'residual footprint':ti,ab,kw OR 'residual footprints':ti,ab,kw OR 'footprint defect':ti,ab,kw OR 'footprint defects':ti,ab,kw OR 'uncovered tuberosity':ti,ab,kw OR 'uncovered tuberosities':ti,ab,kw OR 'uncovered greater tuberosity':ti,ab,kw OR 'exposed greater tuberosity':ti,ab,kw)

**Search Strategy 2 – Imaging-Based Definitions and Structural Parameters (extended)**

('rotator cuff injury'/exp OR 'rotator cuff tear':ti,ab,kw OR 'rotator cuff rupture':ti,ab,kw) AND (massive:ti,ab,kw OR irreparable:ti,ab,kw OR repairability:ti,ab,kw OR reparable:ti,ab,kw) AND ('diagnostic imaging'/exp OR 'magnetic resonance imaging'/exp OR imaging:ti,ab,kw OR radiolog*:ti,ab,kw OR mri:ti,ab,kw OR 'magnetic resonance':ti,ab,kw) AND ( criteria:ti,ab,kw OR definition*:ti,ab,kw OR classification*:ti,ab,kw OR parameter*:ti,ab,kw OR predictor*:ti,ab,kw OR grading:ti,ab,kw OR measurement:ti,ab,kw OR quantitat*:ti,ab,kw OR reliability:ti,ab,kw OR reproducibility:ti,ab,kw OR interobserver:ti,ab,kw OR intraobserver:ti,ab,kw OR cutoff:ti,ab,kw OR threshold:ti,ab,kw OR goutallier:ti,ab,kw OR patte:ti,ab,kw OR 'fatty infiltration':ti,ab,kw OR retraction:ti,ab,kw OR 'muscle atrophy':ti,ab,kw OR 'tangent sign':ti,ab,kw OR 'occupation ratio':ti,ab,kw OR 'acromiohumeral distance':ti,ab,kw OR 'number of tendons':ti,ab,kw OR 'tendon involvement':ti,ab,kw OR outcome*:ti,ab,kw OR function*:ti,ab,kw OR prognosis:ti,ab,kw OR healing:ti,ab,kw OR retear:ti,ab,kw OR postoperative:ti,ab,kw OR footprint*:ti,ab,kw OR 'greater tuberosity exposure':ti,ab,kw OR 'tuberosity exposure':ti,ab,kw OR 'footprint exposure':ti,ab,kw OR 'exposed footprint':ti,ab,kw OR 'exposed footprints':ti,ab,kw OR 'uncovered footprint':ti,ab,kw OR 'uncovered footprints':ti,ab,kw OR 'bare footprint':ti,ab,kw OR 'bare footprints':ti,ab,kw OR 'bare tuberosity':ti,ab,kw OR 'bare tuberosities':ti,ab,kw OR 'tendon coverage':ti,ab,kw OR 'footprint coverage':ti,ab,kw OR 'residual footprint':ti,ab,kw OR 'residual footprints':ti,ab,kw OR 'footprint defect':ti,ab,kw OR 'footprint defects':ti,ab,kw OR 'uncovered tuberosity':ti,ab,kw OR 'uncovered tuberosities':ti,ab,kw OR 'uncovered greater tuberosity':ti,ab,kw OR 'exposed greater tuberosity':ti,ab,kw)

*Database 3: Cochrane Central Register of Controlled Trials (CENTRAL) – initial search (17 February 2026)*

**Platform:** Wiley

**Coverage:** From inception to 17 February 2026

**Date of last search:** 17 February 2026

**Limits applied:** None

**Results:** Search 1 = 7; Search 2 = 398

**Search Strategy 1 – Greater Tuberosity Exposure/Coverage**

("rotator cuff tear*" OR "rotator cuff rupture*") AND ("greater tuberosity" OR "tuberculum majus") AND (exposure OR coverage OR uncover* OR footprint)

**Search Strategy 2 – Imaging-Based Definitions and Structural Parameters**

("rotator cuff tear*" OR "rotator cuff rupture*") AND (massive OR irreparable OR repairability OR reparable) AND (imaging OR MRI OR "magnetic resonance") AND (criteria OR definition* OR classification* OR parameter* OR grading OR measurement OR reliability OR Goutallier OR Patte OR "fatty infiltration" OR retraction OR "muscle atrophy" OR outcome* OR prognosis OR healing OR retear)

*Database 3 (update search): Cochrane Central Register of Controlled Trials (CENTRAL)*

**Platform:** Wiley

**Coverage:** From inception to 9 October 2026 (update search; initial search: inception to 17 February 2026)

**Date of last search:** 9 October 2026

**Limits applied:** None

**Results:** Search 1 = 21; Search 2 = 442

**Note:** Entered in the general search field (Search All Text); field codes, the MeSH term and the NEXT operator were omitted because the platform did not accept them. Only records from CENTRAL (trials) were counted.

**Search Strategy 1 – Greater Tuberosity Exposure/Coverage (extended)**

"rotator cuff" AND ((("greater tuberosity" OR "tuberculum majus") AND (exposure OR coverage OR uncover* OR footprint*)) OR "greater tuberosity exposure" OR "tuberosity exposure" OR "footprint exposure" OR "exposed footprint" OR "exposed footprints" OR "uncovered footprint" OR "uncovered footprints" OR "bare footprint" OR "bare footprints" OR "bare tuberosity" OR "bare tuberosities" OR "tendon coverage" OR "footprint coverage" OR "residual footprint" OR "residual footprints" OR "footprint defect" OR "footprint defects" OR "uncovered tuberosity" OR "uncovered tuberosities" OR "uncovered greater tuberosity" OR "exposed greater tuberosity")

**Search Strategy 2 – Imaging-Based Definitions and Structural Parameters (extended)**

("rotator cuff tear*" OR "rotator cuff rupture*") AND (massive OR irreparable OR repairability OR reparable) AND (imaging OR MRI OR "magnetic resonance") AND (criteria OR definition* OR classification* OR parameter* OR grading OR measurement OR reliability OR Goutallier OR Patte OR "fatty infiltration" OR retraction OR "muscle atrophy" OR outcome* OR prognosis OR healing OR retear OR footprint* OR "greater tuberosity exposure" OR "tuberosity exposure" OR "footprint exposure" OR "exposed footprint" OR "exposed footprints" OR "uncovered footprint" OR "uncovered footprints" OR "bare footprint" OR "bare footprints" OR "bare tuberosity" OR "bare tuberosities" OR "tendon coverage" OR "footprint coverage" OR "residual footprint" OR "residual footprints" OR "footprint defect" OR "footprint defects" OR "uncovered tuberosity" OR "uncovered tuberosities" OR "uncovered greater tuberosity" OR "exposed greater tuberosity")

*Database 4: Scopus (citation searching)*

**Platform:** Scopus

**Date of last search:** 11 October 2026

**Seed publications:** Schumaier et al. 2020 (forward: 85 records; backward: 42 records); Jo et al. 2025 (forward: 4 records; backward: 26 records)

**Results: 157 records in total (115 after removal of 42 duplicates; 5 of these could not be imported into EndNote and were screened manually)**

| **Author** | **Title** | **Primary reason for exclusion** |
| --- | --- | --- |
| Alharairi 2026 | Exploring the level of association between rotator cuff tears and acromiohumeral distance: a systematic review | Review / expert opinion / consensus |
| Altuntas 2025 | Association between acromial morphology and tear severity in arthroscopically confirmed rotator cuff tears | Other non-definitional imaging study |
| Araya-Quintanilla 2021 | Association between type of rotator cuff tear and functional outcomes in patients with massive and irreparable rotator cuff tear: A pre-post intervention study | Not MRCT-specific |
| Arino 2026 | Quantitative evaluation of fatty change in rotator cuff muscles using the Otsu thresholding method | Imaging methodology only |
| Bedi 2010 | Massive tears of the rotator cuff | Review / expert opinion / consensus |
|  |  |  |
| Ben 2025 | Improved Acromiohumeral Distance Independently Predicts Better Outcomes After Arthroscopic Superior Capsular Reconstruction Graft Tears | Procedure / postoperative-focused |
| Brolin 2017 | Classifications in Brief: Hamada Classification of Massive Rotator Cuff Tears | Non-MRI–based study |
| Buess 2011 | Outcome of arthroscopic rotator cuff repair in large tears: the exposed footprint | Non-MRI–based study |
| Castricini 2014 | Irreparable rotator cuff tears: a novel classification system | Not MRCT-specific |
| Charousset 2005 | Accuracy of CT arthrography in the assessment of tears of the rotator cuff | Non-MRI–based study |
|  |  |  |
| Chitkara 2016 | Rotator Cuff Fatty Infiltration Are Coronal Images More Helpful for Characterization than Sagittal Images? | Imaging methodology only |
| Daggett 2015 | Correlation between glenoid inclination and critical shoulder angle: a radiographic and computed tomography study | Non-MRI–based study |
| Daskareh 2025 | Comparing 3D MERGE MRI and 2D MRI for Rotator Cuff Tear Evaluation and Surgical Planning; A Cross-Sectional Prospective Study | Imaging methodology only |
| Denard 2023 | An Algorithmic Approach to the Surgical Management of Massive Rotator Cuff Tears Based on Imaging, Function, and Repairability | Review / expert opinion / consensus |
| Denard et al. 2015 | Pseudoparalysis From a Massive Rotator Cuff Tear Is Reliably Reversed With an Arthroscopic Rotator Cuff Repair in Patients Without Preoperative Glenohumeral Arthritis | Other non-definitional imaging study |
| Farazdaghi 2021 | Comparison between partial and full coverage repair in massive rotator cuff tears. A minimum five year follow-up | Procedure / postoperative-focused |
| Fukuta 2016 | Tendon retraction with rotator cuff tear causes a decrease in cross-sectional area of the supraspinatus muscle on magnetic resonance imaging | Imaging methodology only |
| Guadagno 2023 | Massive rotator cuff tears: algorithmic approach to surgical treatment | Review / expert opinion / consensus |
| Guo 2024 | The Suprascapular Artery as a Reference for the Evaluation of Supraspinatus Fatty Infiltration on Magnetic Resonance Imaging | Imaging methodology only |
| Gupta 2024 | Medial scapular body (MSB) Goutallier classification: MRI-based reliability and validity of evaluation of the Goutallier classification for grading fatty infiltration of the rotator cuff | Imaging methodology only |
| Gyftopoulos 2016 | Rotator cuff tear shape characterization: a comparison of two-dimensional imaging and three-dimensional magnetic resonance reconstructions | Imaging methodology only |
| Harada 2021 | Combination of risk factors affecting retear after arthroscopic rotator cuff repair: a decision tree analysis | Not MRCT-specific |
| Hsu 2024 | Factors associated with reparability of rotator cuff tears: a systematic review and meta-analysis | Review / expert opinion / consensus |
| Hu 2026 | Magnetic resonance imaging-based fat infiltration grading improves reparability prediction in massive rotator cuff tears | Imaging methodology only |
| Iijima 2017 | Differences in fatty degeneration of rotator cuff muscles at different sites, as quantified by T2 mapping | Imaging methodology only |
| Jeon 2021 | Significance of the acromiohumeral distance on stress radiography for predicting healing and function after arthroscopic repair of massive rotator cuff tears | Non-MRI–based study |
| Jeong 2022 | Does Complete Footprint Coverage Affect Outcomes After Conventional Arthroscopic Repair of Large-Sized Rotator Cuff Tears? | Not MRCT-specific |
| Jung 2020 | Diagnostic value of the axial view of magnetic resonance imaging to identify two-dimensional shapes of full-thickness rotator cuff tears | Imaging methodology only |
| Kim I.B. 2018 | Prediction of the Irreparability of Rotator Cuff Tears | Not MRCT-specific |
| Kim M.S. 2022 | Anteroposterior and Lateral Coverage of the Acromion: Prediction of the Rotator Cuff Tear and Tear Size | Non-MRI–based study |
| Kim S.C. 2022 | Preoperative rotator cuff tendon integrity, tear size, and muscle atrophy and fatty infiltration are associated with structural outcomes of arthroscopic revision rotator cuff repair | Not MRCT-specific |
| Kissenberth 2014 | A positive tangent sign predicts the repairability of rotator cuff tears | Not MRCT-specific |
| Koh 2014 | Preoperative factors affecting footprint coverage in rotator cuff repair | Not MRCT-specific |
| Kuan/Shih/Su 2023 | Definition of irreparable rotator cuff tear: a scoping review of prospective surgical therapeutic trials to evaluate current practice | Review / expert opinion / consensus |
| Lädermann 2015 | Massive rotator cuff tears: definition and treatment | Review / expert opinion / consensus |
| Lädermann 2016 | Classification of full-thickness rotator cuff lesions: a review | Review / expert opinion / consensus |
| Lee 2018 | Can a Single Sagittal Magnetic Resonance Imaging Slice Represent Whole Fatty Infiltration in Chronic Rotator Cuff Tears at the Supraspinatus? | Imaging methodology only |
| Liu 2022 | Advantages of 3-dimensional Measurements for Supraspinatus Intramuscular Fatty Evaluation in Patients With Medium to Massive Rotator Cuff Tears: Comparison With a Single Sagittal Slice | Imaging methodology only |
| Matsuki 2014 | Quantitative evaluation of fatty degeneration of the supraspinatus and infraspinatus muscles using T2 mapping | Imaging methodology only |
| Matsumura 2017 | Quantitative assessment of fatty infiltration and muscle volume of the rotator cuff muscles using 3-dimensional 2-point Dixon magnetic resonance imaging | Imaging methodology only |
| Millett 2014 | Posterosuperior rotator cuff tears: classification, pattern recognition, and treatment | Review / expert opinion / consensus |
| Mirzayan 2020 | Is there a difference in the acromiohumeral distances measured on radiographic and magnetic resonance images of the same shoulder with a massive rotator cuff tear? | Imaging methodology only |
| Murthy 2025 | Imaging of Reparable and Irreparable Rotator Cuff Tears | Review / expert opinion / consensus |
| Park I. 2020 | A Novel Reparability Assessment Scoring System for Full-Thickness Rotator Cuff Tears | Not MRCT-specific |
| Quinlan 2021 | Footprint size matters: wider coronal greater tuberosity width is associated with increased rates of healing after rotator cuff repair | Not MRCT-specific |
| Reddy 2024 | Pseudoparesis and pseudoparalysis in the setting of massive irreparable rotator cuff tear: demographic, anatomic, and radiographic risk factors | Other non-definitional imaging study |
| Samim 2018 | Postoperative MRI of Massive Rotator Cuff Tears | Review / expert opinion / consensus |
| Schumaier 2020 | Defining massive rotator cuff tears: a Delphi consensus study | Review / expert opinion / consensus |
| Sela 2015 | Rotator cuff tears: correlation between geometric tear patterns on MRI and arthroscopy and pre- and postoperative clinical findings | Not MRCT-specific |
| Shibayama 2022 | Relationship between preoperative size of rotator cuff tears measured using radial-slice magnetic resonance images and postoperative rotator cuff integrity: a prospective case-control study | Not MRCT-specific |
| Shin 2025 | The Prevalence of Tear Patterns and Their Effects on Tendon Healing After Arthroscopic Surgery in Patients With Full-Thickness Rotator Cuff Tears | Not MRCT-specific |
| Sheth & Shah 2023 | Massive and Irreparable Rotator Cuff Tears: A Review of Current Definitions and Concepts | Review / expert opinion / consensus |
| Suarez Jimenez 2026 | Arthroscopic double row repair of massive posterosuperior tear: healing and functional outcomes | Procedure / postoperative-focused |
| Sugaya 2007 | Repair integrity and functional outcome after arthroscopic double-row rotator cuff repair. A prospective outcome study | Not MRCT-specific |
| Takeuchi 2021 | Stump classification was correlated with retear in the suture-bridge and double-row repair techniques for arthroscopic rotator cuff repair | Not MRCT-specific |
|  |  |  |
| Taniguchi 2017 | Translation of the humeral head scale is associated with success of rotator cuff repair for large-massive tears | Non-MRI–based study |
| Taniguchi 2018 | A new scale measuring translation of the humeral head as a prognostic factor for the treatment of large and massive rotator cuff tears | Non-MRI–based study |
| Tunalı 2022 | Are critical shoulder angle and acromion index correlated to the size of a rotator cuff tear | Non-MRI–based study |
| van der Zwaal 2012 | Preoperative agreement on the geometric classification and 2-dimensional measurement of rotator cuff tears based on magnetic resonance arthrography | Imaging methodology only |
| Werthel 2022 | Three-dimensional muscle loss assessment: a novel computed tomography-based quantitative method to evaluate rotator cuff muscle fatty infiltration | Non-MRI–based study |
| Wi 2026 | Biceps augmentation to improve the footprint coverage and functional outcomes in the treatment of large to massive rotator cuff tears | Procedure / postoperative-focused |
| Yang 2026 | Development of a nomogram prediction model for re-tear after arthroscopic repair of massive rotator cuff tears | Not published in English |
| Zhang 2025 | A new section of shoulder magnetic resonance imaging (MRI) used to assess the fat infiltration of the rotator cuff muscles | Imaging methodology only |

Supplementary Table S1: Full-text articles excluded after eligibility assessment and primary reasons for exclusion

| **Parameter** | **Imaging Modality** | **Imaging Plane** | **Anatomical Landmarks** | **Measurement Type** | **Representative Thresholds / Cut-offs** | **Key References** | **Level of Standardization** |
| --- | --- | --- | --- | --- | --- | --- | --- |
| Greater tuberosity exposure (GTE) | MRI / intraoperative assessment | Sagittal (conceptual) | GT footprint, exposed tuberosity | Relative (% exposure) | ≥67% GTE (conceptual threshold) | Schumaier 2020 (6); Jo 2025 (25); Tanaka 2021 (9) (indirect footprint score only) | None – no standardized imaging methodology or validated thresholds |
| Residual tendon attachment (footprint zones) | MRI | Sagittal oblique | Footprint divided into five zones (crest of lesser tuberosity, lesser tuberosity, superior, middle, and inferior facet) | Semi-quantitative (score 0–5) | None | Tanaka 2021 (9) | Low – own scoring system, reliability reported, no thresholds; does not measure GTE |
| Tear size (ML/AP) | MRI | Coronal oblique (ML); sagittal oblique (AP) | Tendon edge, GT footprint, tear margins | Quantitative (mm/cm) | >5 cm; ML ≥36 mm; AP ≥22–32 mm | Yoo 2009 (37); Kim S.J. 2016 (27); Kuptniratsaikul 2019 (28); Itoigawa 2026 (11); Yoshimura 2026 (12) | Moderate |
| Tendon involvement | MRI / arthroscopy | Multiplanar | SSP, ISP, SSC, TM tendons | Qualitative / semi-quantitative | ≥2 tendons involved | Ok 2017 (31); Furuhata 2022 (21); Ying 2025 (36) | Moderate |
| Tendon retraction | MRI | Primarily coronal | Footprint, humeral head, glenoid rim | Semi-quantitative (Patte classification) | Patte grade 3; modified Patte stage III | Kim J.Y. 2017 (26); Guo 2020 (22); Park 2020 (32) | Moderate |
| Deep-layer shift angle and medial retraction | MRI | Sagittal (shift angle); coronal (medial retraction) | Fitted circle of the humeral head, posterior edge of the long head of the biceps, most anterior deep-layer attachment; lateral cartilage margin and cuff stump | Quantitative (° and mm) | Shift angle 98.0°; medial retraction 29.8 mm (ROC-derived) | Yoshimura 2026 (12) | Low – single-centre derivation, no external validation |
| Fatty infiltration (qualitative) | MRI | Sagittal “Y-view” | Scapular spine–body junction, muscle belly | Ordinal grading (Goutallier) | Goutallier ≥2 or ≥3 | Kim J.Y. 2017 (26); Ohzono 2017 (30); Sheean 2018 (33); Gallego 2026 (10); Itoigawa 2026 (11) | Moderate (slice-selection dependent) |
| Fatty infiltration (quantitative) | MRI | Sagittal, medial sagittal, or 3D sequences | Muscle ROI, fat reference ROI | Quantitative (% fat fraction / T2 mapping) | Fat fraction >33.2%; SSP T2 ≥71.8 ms; ISP T2 ≥63.1 ms | Xie 2025 (35); Iijima 2019 (24) | Moderate to high |
| Muscle atrophy / tangent sign | MRI | Sagittal “Y-view” | Coracoid, scapular spine, supraspinatus contour | Qualitative / semi-quantitative | Positive tangent sign; occupation ratio <0.46 | Kim S.J. 2016 (27); Ohzono 2017 (30); Gallego 2026 (10); Yoshimura 2026 (12) | Moderate |
| Acromiohumeral distance (AHD/AHI) | Radiography and/or MRI | AP radiograph; coronal MRI | Inferior acromion, superior humeral head | Quantitative (mm) | AHD <7 mm; AHI ≤6 mm; AHD <4 mm | Sheean 2018 (33); Furuhata 2022 (21); Guo 2020 (22); Chen 2025 (8) | Moderate |
| Inferior glenohumeral distance (IGHD) | MRI / radiography | Coronal / AP | Inferior glenoid, inferior humeral head | Quantitative (mm) | IGHD >5 mm; ≥8 mm | Kuptniratsaikul 2019 (28); Dwyer 2015 (18); Park 2020 (32) | Low to moderate |
| Tear geometry / pattern | MRI and/or arthroscopy | Coronal and sagittal | Tear edges, footprint, humeral head | Classification-based | Type III combined ML/AP extension | Davidson & Burkhart 2010 (4); Ying 2025 (36) | Moderate |
| Tendon stump signal intensity (stump classification) | MRI | Oblique coronal T2-weighted fat-suppressed | Circular ROI at the tendon stump (5–8 mm from the stump edge) and deltoid muscle | Quantitative (signal-intensity ratio, three types) | Stump/deltoid ratio <0.8 (type 1); 0.8–1.3 (type 2); >1.3 (type 3) | Ben 2024 (7) | Low to moderate – sequence-dependent; reliability reported |

Supplementary Table S2: Overview of MRI-derived parameters used in the structural characterization of massive rotator cuff tears (MRCTs), including imaging methodology, measurement approaches, representative thresholds, and degree of methodological standardization. Greater tuberosity exposure (GTE) is included as a conceptual parameter but currently lacks a validated MRI-based measurement framework.

**Abbreviations:** AHD, acromiohumeral distance; AHI, acromiohumeral interval; AP, anteroposterior; CSA, critical shoulder angle; FI, fatty infiltration; GT, greater tuberosity; GTE, greater tuberosity exposure; IGHD, inferior glenohumeral distance; ISP, infraspinatus tendon; ML, mediolateral; MRI, magnetic resonance imaging; MRCT, massive rotator cuff tear; ROI, region of interest; SSC, subscapularis tendon; SSP, supraspinatus tendon; TM, teres minor tendon.

**Supplementary Table S3: Individual domain-level risk-of-bias judgments for the 22 prognostic studies assessed using the Quality In Prognosis Studies (QUIPS) tool. Domains were rated as low, moderate, or high risk of bias; no overall judgment was derived. For Yoshimura 2026, numerical inconsistencies between the abstract, the results text, and Table 1 of the source publication were noted (extraction was based on Table 1) and are reflected in the rating of prognostic factor measurement.**

| **Study** | **Study Population** | **Study Attrition** | **Prognostic Factor Measurement** | **Outcome Measurement** | **Confounding** | **Statistical Analysis** |
| --- | --- | --- | --- | --- | --- | --- |
| Bedeir 2024 | Moderate | Moderate | Moderate | Low | Moderate | Moderate |
| Ben 2024 | Moderate | Moderate | Low | Low | Moderate | Moderate |
| Chen 2025 | Moderate | Moderate | Moderate | Moderate | Moderate | High |
| Cho & Rhee 2009 | Moderate | Moderate | Moderate | Low | High | Moderate |
| Di Benedetto 2019 | Moderate | Moderate | Moderate | Low | Moderate | Moderate |
| Dwyer 2015 | Moderate | Low | Moderate | Low | High | Moderate |
| Ernstbrunner 2020 | Moderate | Low | Moderate | Moderate | High | Moderate |
| Gallego 2026 | Moderate | Moderate | Moderate | Low | Moderate | Moderate |
| Guo 2020 | Moderate | Moderate | Low | Low | Moderate | Low |
| Guo 2023 | Moderate | Moderate | Moderate | Moderate | High | Moderate |
| Iijima 2019 | Moderate | Low | Moderate | Low | Moderate | Moderate |
| Itoigawa 2026 | Moderate | Low | Moderate | Moderate | Moderate | Moderate |
| Kim J.Y. 2017 | Moderate | Low | Moderate | Low | Moderate | Moderate |
| Kim S.J. 2016 | Moderate | Low | Moderate | Low | Moderate | Moderate |
| Kuptniratsaikul 2019 | Moderate | Low | Moderate | Low | Moderate | Moderate |
| Ohzono 2017 | Moderate | Moderate | Moderate | Low | Moderate | Moderate |
| Ok 2017 | Moderate | Moderate | Moderate | Moderate | Moderate | Moderate |
| Park S.H. 2020 | Moderate | Low | Moderate | Low | Moderate | Moderate |
| Tanaka 2021 | Moderate | Low | Moderate | Low | High | Moderate |
| Xie 2025 | Moderate | Low | Moderate | Low | Moderate | Moderate |
| Ying 2025 | Moderate | Moderate | Moderate | Moderate | Moderate | Moderate |
| Yoshimura 2026 | Moderate | Low | Moderate | Moderate | Moderate | Moderate |
|  |  |  |  |  |  |  |
| **Legend:** | Low |  | Moderate |  | High |  |

**Supplementary Table S4: List of abbreviations used throughout the manuscript, tables, figures, and supplementary materials.**

| **Abbreviation** | **Definition** |
| --- | --- |
| AHD | Acromiohumeral distance |
| AHI | Acromiohumeral interval |
| AI | Acromial index |
| AP | Anteroposterior |
| ASES | American Shoulder and Elbow Surgeons score |
| CENTRAL | Cochrane Central Register of Controlled Trials |
| CSA | Critical shoulder angle |
| CT | Computed tomography |
| COTD | Coronal-oblique tendon displacement |
| FI | Fatty infiltration |
|  |  |
| GFDI | Global fatty degeneration index |
| GT | Greater tuberosity |
| GTE | Greater tuberosity exposure |
| IGHD | Inferior glenohumeral distance |
| ISP | Infraspinatus tendon |
| JOA | Japanese Orthopaedic Association score |
| L/W | Length/width |
| LoE | Level of evidence |
| MeSH | Medical Subject Headings |
| ML | Mediolateral |
| MR | Magnetic resonance |
| MRI | Magnetic resonance imaging |
| MRCT | Massive rotator cuff tear |
| nCSA | Normalized critical shoulder angle |
|  |  |
| PRISMA | Preferred Reporting Items for Systematic Reviews and Meta-Analyses |
| PRISMA-S | Preferred Reporting Items for Systematic Reviews and Meta-Analyses extension for literature searches |
| QUADAS-2 | Quality Assessment of Diagnostic Accuracy Studies 2 |
| QUIPS | Quality In Prognosis Studies |
| ROI | Region of interest |
|  |  |
| SSC | Subscapularis tendon |
| SSP | Supraspinatus tendon |
| SOTD | Sagittal-oblique tendon displacement |
| SWE | Shear wave elastography |
| TM | Teres minor tendon |
| UCLA | University of California, Los Angeles shoulder score |
| UMI | Upward migration index |
